# The Choquet integral of log-convex functions

**DOI:** 10.1186/s13660-018-1803-y

**Published:** 2018-08-14

**Authors:** Hongxia Wang

**Affiliations:** 1 College of Statistics, Henan University of Economics and Law, Zhengzhou, China; 2 Analysis and Research Center on Education and Statistic Data of Henan Province, Zhengzhou, China

**Keywords:** Choquet integral, Log-convex function, Inequality

## Abstract

In this paper we investigate the upper bound and the lower bound of the Choquet integral for log-convex functions. Firstly, for a monotone log-convex function, we state the similar Hadamard inequality of the Choquet integral in the framework of distorted measure. Secondly, we estimate the upper bound of the Choquet integral for a general log-convex function, respectively, in the case of distorted Lebesgue measure and in the non-additive measure. Finally, we present Jensen’s inequality of the Choquet integral for log-convex functions, which can be used to estimate the lower bound of this kind when the non-additive measure is concave. We provide some examples in the framework of the distorted Lebesgue measure to illustrate all the results.

## Introduction

It is well known that the concept of non-additive measure [1] can be used to deal with some uncertainty phenomena which cannot be easily modeled by using additive measure, and the Choquet integral, which also covers the classical Lebesgue integral, is one kind of nonlinear expectations. Many authors developed the Choquet theory with its applications in many areas such as multicriteria decision making, risk measuring, option pricing, and so on. Readers may refer to the references [2–17]. Here we specially mention that Mesiar et al. [9] discussed the integral inequalities known for the Lebesgue integral in the framework of the Choquet integral.

A strong property of convexity is log-convexity. It is known that every log-convex function is also convex. In many applications, assumptions about the log-convexity of a probability distribution allow just enough special structure to yield a workable theory. The log-convexity (log-concavity) of probability densities and their integrals has interesting qualitative implications in many areas of economics, in political science, in biology, and in industrial engineering [18].

Thus the study of the Choquet integral of log-convex functions is an important and interesting topic for further research. It is well known that the Hadamard inequality is a famous and important result, which provides the upper (lower) bound for the mean value of a log-convex (log-concave) function. Abbaszadeh et al. [19] studied the Sugeno fuzzy integral of log-convex functions and showed that the Hadamard inequality is not valid for this kind of Sugeno fuzzy integral. Motivated by [19], we naturally wonder whether the Hadamard inequality still holds for the Choquet integral. If the Hadamard inequality is not valid, then it is necessary to estimate the upper bound and the lower bound of the Choquet integral for log-convex functions.

In this paper, we shall study the upper bound and the lower bound of the Choquet integral for log-convex functions. The rest of this paper is organized as follows. Section 2 is for preliminaries and notations of the non-additive measure, the Choquet integral, and log-convex function that will be used later. In Sect. 3, the main results are shown. Firstly, we shall point out that the Hadamard inequality is not valid in the framework of distorted Lebesgue measure and shall present a similar Hadamard inequality for monotone log-convex functions. Secondly, the upper bound of the Choquet integral for the general log-convex function is presented. Finally, we shall investigate Jensen’s inequality of the Choquet integral for a log-convex function, which can be used to estimate the lower bound of this kind. At the end of the paper, some conclusions are drawn and some problems for further investigation are suggested.

## Preliminaries

Throughout the paper, assume that $(X,\mathcal{F})$ is a measurable space and ($\mathbf{R}^{+}$) **R** is the set of all (nonnegative) real numbers.

We first recall some concepts and some elementary results of capacity and the Choquet integral [1, 2].

### Definition 2.1

A set function $\mu : \mathcal{F} \rightarrow \mathbf{R}^{+}$ is called a non-additive measure if it satisfies

1. $\mu (\emptyset )=0$;
2. $\mu (A)\leq \mu (B)$ for any $A\subseteq B$ and $A,B\in \mathcal{F}$.

Given a non-additive measure *μ* on $(X,\mathcal{F})$, with $\mu (X)$ finite (i.e., $\mu (X)<\infty $), we define its conjugate or dual as the measure *μ̄* defined as follows: $\bar{\mu }(A)= \mu (X)-\mu (A^{c})$ for all $A\in \mathcal{F}$. Here and in the sequel $A^{c}$ denotes the complement set of *A*. When a measure *μ* is additive, it holds that $\mu (A)=\bar{\mu }(A)$.

The non-additive measure *μ* is called concave if

$$ \mu (A\cup B)+\mu (A\cap B)\leq \mu (A)+\mu (B) $$

for all $A,B\in \mathcal{F}$. In the literature the concave non-additive measure is known as submodular or 2-alternating non-additive measure. If the above inequality is reversed, *μ* is called convex. Similarly, convexity is called supermodularity or 2-monotonicity, too.

First note that the Lebesgue measure *λ* for an interval $[a, b]$ is defined by $\lambda ([a, b]) = b-a$, and that given a distortion function *m*, which is increasing (or non-decreasing) and such that $m(0)=0$, the measure $\mu (A)=m(\lambda (A))$ is a distorted Lebesgue measure. We denote a Lebesgue measure with distortion *m* by $\mu =\mu_{m}$. It is not difficult to know that $\mu_{m}$ is concave (convex) if *m* is a concave (convex) function.

The family of all the nonnegative, measurable functions $f: (X, \mathcal{F})\rightarrow (\mathbf{R}^{+}, \mathcal{B}(\mathbf{R}^{+}))$ is denoted as $L_{+}^{\infty }$, where $\mathcal{B}(\mathbf{R}^{+})$ is the Borel *σ*-field of $\mathbf{R}^{+}$. The concept of the integral with respect to a non-additive measure was introduced by Choquet [1].

### Definition 2.2

Let $f\in L_{+}^{\infty } $. The Choquet integral of *f* with respect to non-additive measure *μ* on $A\in \mathcal{F}$ is defined by

1 $$ (C) \int_{A} f \,d\mu = \int_{0}^{+\infty }\mu \bigl(\bigl\{ x: f(x)\geq t\bigr\} \cap A\bigr)\,dt, $$

where the integrals on the right-hand side are taken in the sense of Lebesgue.

Instead of $(C)\int_{X} f \,d\mu $, we shall write $(C)\int f \,d\mu $. If $(C)\int f \,d\mu <\infty $, we say that *f* is Choquet integrable. For $f\in L_{+}^{\infty }$, we write

$$ L_{C}^{+}(\mu )= \biggl\{ f: (C){ \int f \,d\mu }< \infty \biggr\} . $$

The subsequent lemma summarizes the basic properties of Choquet integrals [2].

### Lemma 2.1

*Assume that* *f*, $g\in L_{C}^{+}(\mu )$.

1. $(C)\int 1_{A}\,d\mu =\mu (A), A\in \mathcal{F}$.
2. (*Positive homogeneity*) *For all* $\lambda \in \mathbf{R}^{+}$, *we have* $(C) \int \lambda f\,d\mu =\lambda \cdot (C)\int f \,d\mu $.
3. (*Translation invariance*) *For all* $c\in \mathbf{R}$, *we have* $(C) \int (f+c)\,d\mu =(C)\int f \,d\mu +c$.
4. (*Monotonicity in the integrand*) *If* $f\leq g$, *then we have*
  $$ (C) \int f \,d\mu \leq (C) \int g \,d\mu ; $$
  (*Monotonicity in the set function*) *If* $\mu \leq \nu $, *then we have* $(C) \int f \,d\mu \leq (C)\int f \,d\nu $.
5. (*Subadditivity*) *If* *μ* *is concave*, *then*
  $$ (C) \int (f+g)\,d\mu \leq (C) \int f \,d\mu +(C) \int g \,d\mu ; $$
  (*Superadditivity*) *If* *μ* *is convex*, *then*
  $$ (C) \int (f+g)\,d\mu \geq (C) \int f \,d\mu +(C) \int g \,d\mu . $$
6. (*Comonotonic additivity*) *If* *f* *and* *g* *are comonotonic*, *then*
  $$ (C) \int (f+g)\,d\mu =(C) \int f \,d\mu +(C) \int g \,d\mu , $$
  *where we say that* *f* *and* *g* *are comonotonic*, *if for any* $x,x'\in X$, *then*
  $$ \bigl(f(x)-f\bigl(x'\bigr)\bigr) \bigl(g(x)-g\bigl(x' \bigr)\bigr)\geq 0. $$

We review now the excellent results from the article [20], which permits us to compute the Choquet integral when the non-additive measure is a distorted Lebesgue measure.

### Lemma 2.2

*Let* *f* *be a nonnegative and measurable function on* $\mathbf{R}^{+}$ *and* $\mu =\mu_{m}$ *be a distorted Lebesgue measure*. *And assume that* $m(x)$ *and* $f(x)$ *are both continuously differentiable*. *When* *f* *is an increasing* (*non*-*decreasing*) *function on* $\mathbf{R}^{+}$, *the Choquet integral of* *f* *with respect to* $\mu_{m}$ *on* $[0,t]$ *is represented as*

2 $$ (C) \int_{[0,t]} f \,d\mu_{m}= \int_{0}^{t}m'(t-x)f(x)\,dx; $$

*however*, *when* *f* *is a decreasing* (*non*-*increasing*) *function on* $\mathbf{R}^{+}$, *the Choquet integral of* *f* *is*

3 $$ (C) \int_{[0,t]} f \,d\mu_{m}= \int_{0}^{t}m'(x)f(x)\,dx. $$

The following lemma, about Jensen’s inequality of the Choquet integral, comes from [9, 15].

### Lemma 2.3

*Assume that* $f\in L_{C}^{+}(\mu )$ *and the non*-*additive measure* *μ* *is concave on* $(X,\mathcal{F})$. *If* $\Phi : \mathbf{R}^{+}\rightarrow \mathbf{R}^{+}$ *is a convex function*, *then*

4 $$ \Phi \biggl((C) \int f \,d\mu \biggr)\leq (C) \int \Phi \circ f \,d\mu . $$

In the following subsection, we shall list some preliminaries about the log-convex functions. Concerning more definitions and more results of the log-convexity, readers could refer to Zhang and Jiang’s excellent article [21].

Recall the definition of a log-convex (log-concave) function.

### Definition 2.3

Let $f: [a,b]\subseteq \mathbf{R}\rightarrow \mathbf{R}^{+}$. Then *f* is called a log-convex (log-concave) function, if for any $x,y\in [a,b]$ and $\lambda \in [0,1]$ we have

5 $$ f\bigl(\lambda x+(1-\lambda )y\bigr)\leq (\geq ) f(x)^{\lambda } f(y)^{1-\lambda }. $$

If the reverse inequality holds, *f* is termed log-concave.

The logarithmic mean $L(x,y)$ of two positive numbers *x*, *y* is given by

$$ L(x,y)= \textstyle\begin{cases} \frac{x-y}{\ln x-\ln y},& x\neq y, \\ x,& x= y. \end{cases} $$

The following Hadamard inequality provides the upper (lower) bound for the mean value of a measurable and log-convex (log-concave) function $f:[a,b]\rightarrow \mathbf{R}^{+}$ (see [22]):

6 $$ \frac{1}{b-a} \int_{a}^{b} f(x)\,dx\leq (\geq ) L\bigl(f(a),f(b) \bigr). $$

## Main results

In this paper, we shall study the Choquet integral for log-convex (log-concave) functions.

Firstly, we shall investigate whether the Hadamard inequality for the Choquet integral still holds. The following theorem shows that the Hadamard inequality is not valid in the distorted measure theory. However, we can obtain a similar Hadamard inequality under some certain conditions.

Hereafter, let $m(x)$ and $f(x)$ be continuously differentiable.

### Theorem 3.1

1. *Let* *f* *be a positive*, *measurable*, *decreasing*, *and log*-*convex* (*log*-*concave*) *function on* $\mathbf{R}^{+}$ *and* $\mu =\mu_{m}$ *be a distorted Lebesgue measure*. *Then there exists* $\xi \in (0,1)$ *such that*
  7 $$ (C) \int_{[0,1]} f(x)\,d\mu_{m}\leq (\geq )m'( \xi )L\bigl(f(0),f(1)\bigr). $$
2. *Let* *f* *be a positive*, *measurable*, *increasing*, *and log*-*convex* (*log*-*concave*) *function on* $\mathbf{R}^{+}$ *and* $\mu =\mu_{m}$ *be a distorted Lebesgue measure*. *Then there exists* $\theta \in (0,1)$ *such that*
  8 $$ (C) \int_{[0,1]} f(x)\,d\mu_{m}\leq (\geq )m'(1-\theta )L\bigl(f(0),f(1)\bigr). $$

### Proof

(1) Since the log-convexity (log-concavity) of the function *f*, easily we get

$$ \int_{0}^{1}f(x)\,dx\leq (\geq ) L\bigl(f(0),f(1) \bigr) $$

due to the Hadamard inequality. On the other hand, since *f* is non-increasing, we know that

$$ (C) \int_{[0,1]} f(x)\,d\mu_{m}= \int_{0}^{1}m'(x)f(x)\,dx $$

due to Lemma 2.2. Considering that $f(x)>0$ on $[0,1]$, we know that there exists $\xi \in (0,1)$ such that

$$ \int_{0}^{1}m'(x)f(x) \,dx=m'(\xi ) \int_{0}^{1}f(x)\,dx $$

by the mean value theorems for definite integrals. So we have

$$ (C) \int_{[0,1]} f(x)\,d\mu_{m}=m'(\xi ) \int_{0}^{1}f(x)\,dx\leq (\geq )m'( \xi )L\bigl(f(0),f(1)\bigr),\quad \xi \in (0,1), $$

which completes the proof.

(2) In an analogous way as in the proof of (1), we can obtain the desired result. □

### Example 3.1

Taking the positive, increasing, and log-convex function $f(x)=2^{x^{2}-3}$ on $\mathbf{R}^{+}$, easily we get

$$ \int_{0}^{1}f(x)\,dx= \int_{0}^{1}2^{x^{2}-3}\,dx\leq L \bigl(f(0),f(1)\bigr)\approx 0.1800 $$

due to the Hadamard inequality. On the other hand, if we put $g(x)=x^{2}$, then

$$ (C) \int_{[0,1]} f(x)\,d\mu_{g}= \int_{0}^{1}2(1-x)2^{x^{2}-3}\,dx. $$

Since $2^{x^{2}-3}>0$ on $[0,1]$, we know that there exists $\theta \in (0,1)$ such that

$$ \int_{0}^{1}2(1-x)2^{x^{2}-3}\,dx=2(1-\theta ) \int_{0}^{1}2^{x^{2}-3}\,dx $$

by the mean value theorems for definite integrals. So we have

$$ (C) \int_{[0,1]} f(x)\,d\mu_{g}=2(1-\theta ) \int_{0}^{1}2^{x^{2}-3}\,dx \leq 2(1-\theta )L \bigl(f(0),f(1)\bigr),\quad \theta \in (0,1). $$

In fact,

$$ \int_{0}^{1}2^{x^{2}-3}\,dx\approx 0.1610 $$

and

$$ (C) \int_{[0,1]} f(x)\,d\mu_{g}= \int_{0}^{1}2(1-x)2^{x^{2}-3}\,dx\approx 0.1417, $$

so in this case $\theta \approx 0.5599$.

The next theorem is the general case of Theorem 3.1.

### Theorem 3.2

1. *Let* *f* *be a positive*, *measurable*, *decreasing*, *and log*-*convex* (*log*-*concave*) *function on* $\mathbf{R}^{+}$ *and* $\mu =\mu_{m}$ *be a distorted Lebesgue measure*. *For* $[a,b]\subset \mathbf{R}^{+}$, *we know there exists* $\xi \in (0,b-a)$ *such that*
  9 $$ \frac{1}{b-a}(C) \int_{[a,b]} f(x)\,d\mu_{m}\leq (\geq )m'( \xi )L\bigl(f(a),f(b)\bigr). $$
2. *Let* *f* *be a positive*, *measurable*, *increasing and log*-*convex* (*log*-*concave*) *function on* $\mathbf{R}^{+}$ *and* $\mu =\mu_{m}$ *be a distorted Lebesgue measure*. *For* $[a,b]\subset \mathbf{R}^{+}$, *we know there exists* $\theta \in (0,b-a)$ *such that*
  10 $$ \frac{1}{b-a}(C) \int_{[a,b]} f(x)\,d\mu_{m}\leq (\geq )m'(b-a-\theta )L\bigl(f(a),f(b)\bigr). $$

### Proof

(1) We know that

$$ (C) \int_{[a,b]} f(x)\,d\mu_{m}=(C) \int_{[0,b-a]} f(a+x)\,d\mu_{m}. $$

On the one hand, we get

$$ \int_{0}^{b-a}f(a+x)\,dx\leq (\geq ) (b-a)L \bigl(f(a),f(b)\bigr) $$

due to the Hadamard inequality; on the other hand, we know that

$$ (C) \int_{[0,b-a]} f(a+x)\,d\mu_{m}= \int_{0}^{b-a}m'(x)f(a+x)\,dx $$

due to Lemma 2.2. Since $f(a+x)>0$ on $[0,b-a]$, we know that there exists $\xi \in (0,b-a)$ such that

$$ \int_{0}^{b-a}m'(x)f(a+x) \,dx=m'(\xi ) \int_{0}^{b-a}f(a+x)\,dx $$

by the mean value theorems for definite integrals. So we have

$$ \frac{1}{b-a}(C) \int_{[a,b]} f(x)\,d\mu_{m}=\frac{1}{b-a}m'( \xi ) \int _{0}^{b-a}f(a+x)\,dx\leq (\geq )m'( \xi )L\bigl(f(a),f(b)\bigr), $$

where $\xi \in (0,b-a)$.

(2) In an analogous way as in the proof of (1), we can obtain the desired result. Since $f(a+x)$ is decreasing on $[0,b-a]$, we know that

$$ (C) \int_{[0,b-a]} f(a+x)\,d\mu_{m}= \int_{0}^{b-a}m'(b-a-x)f(a+x)\,dx $$

due to Lemma 2.2. Since $f(a+x)>0$ on $[0,b-a]$, we know that there exists $\theta \in (0,b-a)$ such that

$$ \int_{0}^{b-a}m'\bigl[b-a-(a+x) \bigr]f(a+x)\,dx=m'(b-a-\theta ) \int_{0}^{b-a}f(a+x)\,dx $$

by the mean value theorems for definite integrals. So we have

$$\begin{aligned} \frac{1}{b-a}(C) \int_{[a,b]} f(x)\,d\mu_{m} &= \frac{1}{b-a}m'(b-a- \theta ) \int_{0}^{1}f(a+x)\,dx \\ &\leq (\geq ) m'(b-a-\theta )L\bigl(f(a),f(b)\bigr), \end{aligned}$$

where $\theta \in (0,b-a)$. We complete the proof. □

### Example 3.2

Consider the function $f(x)=x2^{x^{2}-2}$ on $\mathbf{R}^{+}$. This function is positive, increasing, and log-convex. Obviously $f(1)=1/2, f(2)=8$. Let $m(x)=x^{2}$. According to Theorem 3.2 (2), there exists $\theta \in (0,1)$ such that

$$ (C) \int_{[1,2]} f(x)\,d\mu_{m}\leq 2(1-\theta )L \bigl(f(1),f(2)\bigr)\approx 5.4100(1- \theta ) $$

due to $L(f(1),f(2))\approx 2.7050$.

In fact, we have

$$\begin{aligned} (C) \int_{[1,2]} f(x)\,d\mu_{m} &= (C) \int_{[0,1]} f(1+x)\,d\mu_{m} \\ &= \int_{0}^{1} m'(1-x)f(1+x)\,dx \\ &= \int_{0}^{1} 2(1-x)f(1+x)\,dx = 2(1-\theta ) \int_{0}^{1} f(1+x)\,dx, \end{aligned}$$

where $\theta \in [0,1]$. Since

$$ \int_{0}^{1} 2(1-x)f(1+x)\,dx= \int_{0}^{1} 2(1-x) (1+x)2^{(1+x)^{2}-2}\,dt \approx 1.4615 $$

and

$$ \int_{0}^{1} f(1+x)\,dx= \int_{0}^{1} (1+x)2^{(1+x)^{2}-2}\,dx\approx 2.5247, $$

we have $\theta \approx 0.7106$.

Observe that Theorems 3.1 and 3.2 are based on the assumption that the log-convex function is monotone. This suggests an open question: Can we find the upper bound of the Choquet integral when the log-convex function is not monotone? In the following we shall present some results concerning this issue.

### Theorem 3.3

*Let* *f* *be a positive*, *measurable*, *and log*-*convex function on* $\mathbf{R}^{+}$ *and* $\mu =\mu_{m}$ *be a distorted Lebesgue measure*. *If* $f(0)\neq f(1)$, *then we have*

11 $$ (C) \int_{[0,1]} f(x)\,d\mu_{m}\leq \int_{0}^{1} m'(1-x)f(0)^{1-x}f(1)^{x} \,dx $$

*for* $f(0)< f(1)$ *and*

12 $$ (C) \int_{[0,1]} f(x)\,d\mu_{m}\leq \int_{0}^{1} m'(x)f(0)^{1-x}f(1)^{x} \,dx $$

*for* $f(0)>f(1)$.

### Proof

Using the log-convexity of *f*, for $x\in [0,1]$, we have

$$ f(x)=f \bigl((1-x)\cdot 0+x\cdot 1 \bigr)\leq f(0)^{1-x}f(1)^{x}. $$

If $f(0)< f(1)$, then $f(0)^{1-x}f(1)^{x}=f(0)\cdot (\frac{f(1)}{f(0)} )^{x}$ is increasing on $\mathbf{R}^{+}$. By (4) of Lemma 2.1 and Lemma 2.2, we frequently have

$$ (C) \int_{[0,1]} f(x)\,d\mu_{m}\leq (C) \int_{[0,1]} f(0)^{1-x}f(1)^{x}\,d\mu_{m} = \int_{0}^{1} m'(1-x)f(0)^{1-x}f(1)^{x}\,dx. $$

Conversely, if $f(0)>f(1)$, then $f(0)^{1-x}f(1)^{x}$ is decreasing on $[0,1]$. We similarly have

$$ (C) \int_{[0,1]} f(x)\,d\mu_{m}\leq (C) \int_{[0,1]} f(0)^{1-x}f(1)^{x}\,d \mu_{g} = \int_{0}^{1} m'(x)f(0)^{1-x}f(1)^{x} \,dx. $$

The proof is completed. □

### Remark 3.1

When $f(0)=f(1)$ in Theorem 3.3, we have $f(x)\leq f(0), x\in [0,1]$. Taking into account (1) and (2) of Lemma 2.1, we get

$$\begin{aligned} (C) \int_{[0,1]} f(x)\,d\mu_{m} \leq & (C) \int_{[0,1]} f(0)\,d\mu_{m}=(C) \int f(0)\cdot 1_{[0,1]}\,d\mu_{m} \\ =&f(0)\cdot m\bigl(\lambda \bigl([0,1]\bigr)\bigr)=f(0)m(1). \end{aligned}$$

In the next theorem, we prove the general case of Theorem 3.3.

### Theorem 3.4

*Let* *f* *be a positive*, *measurable*, *and log*-*convex function on* $\mathbf{R}^{+}$ *and* $\mu =\mu_{m}$ *be a distorted Lebesgue measure*. *If* $f(a)\neq f(b)$, *then we have*

13 $$ (C) \int_{[a,b]} f(x)\,d\mu_{g}\leq \int_{0}^{b-a} m'(b-a-x)f(a)^{\frac{b-a-x}{b-a}}f(b)^{\frac{x}{b-a}} \,dx $$

*for* $f(a)< f(b)$; *and*

14 $$ (C) \int_{[a,b]} f(x)\,d\mu_{g}\leq \int_{0}^{b-a} m'(x)f(a)^{\frac{b-a-x}{b-a}}f(b)^{\frac{x}{b-a}} \,dx $$

*for* $f(a)>f(b)$.

### Proof

We know that

$$ (C) \int_{[a,b]} f(x)\,d\mu_{g}= (C) \int_{[0,b-a]} f(a+x)\,d\mu_{g}. $$

Using the log-convexity of *f*, for $t= \frac{x}{b-a}\in [0,1]$, we have

$$ f(a+x)= f \bigl((1-t) a+t b \bigr)\leq (\geq )f(a)^{1-t}f(b)^{t}. $$

If $f(a)< f(b)$, then $f(a)^{1-t}f(b)^{t}$ is increasing. By (4) of Lemma 2.1 and Lemma 2.2, we frequently have

$$\begin{aligned} (C) \int_{[a,b]} f(x)\,d\mu_{g} =& (C) \int_{[0,b-a]} f(a+x)\,d\mu_{g} \\ \leq & (C) \int_{[0,b-a]} f(a)^{1-t}f(b)^{t}\,d \mu_{g} \\ =& \int_{0}^{b-a} m'(b-a-x)f(a)^{1-t}f(b)^{t} \,dx, \end{aligned}$$

where $t=\frac{x}{b-a}$.

Conversely, if $f(a)>f(b)$, then $f(a)^{1-t}f(b)^{t}$ is decreasing. We similarly have

$$\begin{aligned} (C) \int_{[a,b]} f(x)\,d\mu_{g} \leq (C) \int_{[0,b-a]} f(a)^{1-t}f(b)^{t}\,d \mu_{g}=\int_{0}^{b-a} m'(x)f(a)^{1-t}f(b)^{t} \,dx , \end{aligned}$$

where $t=\frac{x}{b-a}$.

The proof is completed. □

### Remark 3.2

In the case $f(a)=f(b)$ in Theorem 3.4, we can get

$$\begin{aligned} (C) \int_{[a,b]} f(x)\,d\mu_{m} \leq &(C) \int_{[a,b]} f(a)\,d\mu_{m} \\ =&(C) \int f(a)\cdot 1_{[a,b]}\,d\mu_{m} =f(a)\cdot \mu_{m}\bigl([a,b]\bigr) \\ =&f(a)\cdot m(\lambda \bigl([a,b]\bigr)=f(a)\cdot m(b-a) . \end{aligned}$$

### Remark 3.3

We need to point out that Theorems 3.3 and 3.4 hold for the general log-convex function; that is to say, the two theorems do not require that *f* is monotone. Of course, even so, they hold for a monotone log-convex function.

### Example 3.3

Consider the function $f(x)=2^{x^{2}-3}$ on $\mathbf{R}^{+}$ and the distorted Lebesgue measure $\mu =\mu_{m}$, where $m(x)=x^{2}$ in Example 3.1. We have, according to Theorem 3.3,

$$\begin{aligned} (C) \int_{[0,1]} 2^{x^{2}-3}\,d\mu_{g} \leq & (C) \int_{[0,1]}f(0)^{1-x}f(1)^{x}\,d \mu_{g} = (C) \int_{[0,1]} 2^{x-3}\,d\mu_{g} \\ =& \int_{0}^{1}2(1-x)2^{x-3}\,dx \approx 0.1597 . \end{aligned}$$

### Example 3.4

Consider the function $f(x)=x2^{x^{2}-2}$ on $\mathbf{R}^{+}$ and the distorted Lebesgue measure $\mu =\mu_{m}$, where $m(x)=x^{2}$ in Example 3.2. We have, according to Theorem 3.4,

$$\begin{aligned} (C) \int_{[1,2]} x2^{x^{2}-2}\,d\mu_{g} \leq & (C) \int_{[0,1]} f(1)^{1-x}f(2)^{x}\,d \mu_{g} = (C) \int_{[0,1]} 2^{4x-1}\,d\mu_{g} \\ =& \int_{0}^{1}2(1-x)2^{4x-1}\,dt \approx 1.5906 . \end{aligned}$$

In the following, we shall discuss the upper bound of the Choquet integral for the log-convex function in the framework of the general non-additive measure.

### Theorem 3.5

*Let* *f* *be a nonnegative*, *measurable*, *and log*-*convex function on* $\mathbf{R}^{+}$ *and* *μ* *be a non*-*additive measure*. *If* $f(0)\neq f(1)$, *then we have*

15 $$ (C) \int_{[0,1]} f(x)\,d\mu \leq \int_{0}^{+\infty }\mu \bigl([0,1]\cap \bigl\{ x: f(0)^{1-x}f(1)^{x} \geq r \bigr\} \bigr)\,dr. $$

*Furthermore*, *if* $f(0)< f(1)$, *then*

$$ (C) \int_{[0,1]} f(x)\,d\mu \leq \mu \bigl([0,1]\bigr)f(0)+\ln \frac{f(1)}{f(0)} \int _{0}^{1}\mu \bigl([x,1]\bigr)f(0)^{1-x}f(1)^{x} \,dx; $$

*if* $f(0)>f(1)$, *then*

$$ (C) \int_{[0,1]} f(x)\,d\mu \leq \mu \bigl([0,1]\bigr)f(1)-\ln \frac{f(1)}{f(0)} \int _{0}^{1}\mu \bigl([0,x]\bigr)f(0)^{1-x}f(1)^{x} \,dx. $$

### Proof

Using the log-convexity of *f*, for $x\in [0,1]$, we have

$$ f(x)=f \bigl((1-x)\cdot 0+x\cdot 1 \bigr)\leq f(0)^{1-x}f(1)^{x}. $$

Due to (4) of Lemma 2.1, we frequently have

$$\begin{aligned} (C) \int_{[0,1]} f(x)\,d\mu \leq &(C) \int_{[0,1]} f(0)^{1-x}f(1)^{x}\,d\mu \\ =& \int_{0}^{+\infty }\mu \bigl([0,1]\cap \bigl\{ x: f(0)^{1-x}f(1)^{x} \geq r \bigr\} \bigr)\,dr. \end{aligned}$$

For simplicity, we denote $F(x)=f(0)^{1-x}f(1)^{x}$. It is easy to get $F(0)=f(0), F(1)=f(1)$, and $F'(x)=f(0)^{1-x}f(1)^{x}\ln \frac{f(1)}{f(0)}$.

If $f(0)< f(1)$, then we have

$$\begin{aligned} & \int_{0}^{+\infty }\mu \bigl([0,1]\cap \bigl\{ x: F(x)\geq r \bigr\} \bigr)\,dr \\ &\quad = \int_{0}^{+\infty }\mu \bigl([0,1]\cap \bigl\{ x: x\geq F^{-1}(r) \bigr\} \bigr)\,dr\\ &\quad = \int_{0}^{f(0)}\mu \bigl([0,1]\bigr)\,dr+ \int_{f(0)}^{f(1)}\mu \bigl(\bigl[F^{-1}(r),1\bigr]\bigr)\,dr \\ &\quad =\mu \bigl([0,1]\bigr)f(0)+ \int_{0}^{1}\mu \bigl([x,1]\bigr)F'(x)\,dx . \end{aligned}$$

Conversely, if $f(0)>f(1)$, then we have

$$\begin{aligned} & \int_{0}^{+\infty }\mu \bigl([0,1]\cap \bigl\{ x: F(x)\geq r \bigr\} \bigr)\,dr \\ &\quad = \int_{0}^{+\infty }\mu \bigl([0,1]\cap \bigl\{ x: x\geq F^{-1}(r) \bigr\} \bigr)\,dr \\ &\quad = \int_{0}^{f(1)}\mu \bigl([0,1]\bigr)\,dr+ \int_{f(1)}^{f(0)}\mu \bigl(\bigl[0,F^{-1}(r)\bigr]\bigr)\,dr \\ &\quad =\mu \bigl([0,1]\bigr)f(1)-\int_{0}^{1}\mu \bigl([0,x]\bigr)F'(x) \,dx . \end{aligned}$$

The proof is completed. □

### Remark 3.4

Specially, when $\mu =\mu_{m}$ is a distorted Lebesgue measure, Theorem 3.5 still holds.

### Example 3.5

Consider the function $f(x)=2^{x^{2}-3}$ on $\mathbf{R}^{+}$ and the distorted Lebesgue measure $\mu =\mu_{m}$, where $m(x)=x^{2}$, in Examples 3.1 and 3.3. We have $f(0)=1/8$, $f(1)=1/4$, and

$$\begin{aligned} (C) \int_{[0,1]} 2^{x^{2}-3}\,d\mu \leq & (C) \int_{[0,1]} 2^{-3(1-x)}\cdot 2^{-2x}\,d\mu \\ =& (C)\int_{[0,1]} 2^{x-3}\,d\mu \\ =&\int_{0}^{+\infty }m\bigl(\lambda \bigl([0,1]\cap \bigl\{ x:2^{x-3}\geq r \bigr\} \bigr)\bigr)\,dr \\ =& \int_{0}^{+\infty }m\bigl(\lambda \bigl([0,1]\cap \bigl\{ x\geq\log_{2}^{r}+3 \bigr\} \bigr)\bigr)\,dr \\ =& \int_{0}^{1/8}m\bigl(\lambda \bigl([0,1]\bigr)\bigr)\,dr+\int_{1/8}^{1/4}m\bigl(\lambda \bigl(\bigl[\log _{2}^{r}+3,1\bigr]\bigr)\bigr)\,dr \\ =& \frac{1}{8}+ \int_{0}^{1}m\bigl(\lambda \bigl([x,1]\bigr)\bigr) \bigl(2^{x-3}\bigr)'\,dx \\ =& \frac{1}{8}+ \int_{0}^{1}(1-x)^{2}2^{x-3}\ln 2\,d x\approx 0.1597 . \end{aligned}$$

The next theorem is the general case of Theorem 3.5.

### Theorem 3.6

*Let* *f* *be a nonnegative*, *measurable*, *and log*-*convex function on* $\mathbf{R}^{+}$ *and* *μ* *be a non*-*additive measure*. *For* $[a,b]\subset \mathbf{R}^{+}$, *if* $f(a)\neq f(b)$, *then we have*

16 $$ (C) \int_{[a,b]} f(x)\,d\mu \leq \int_{0}^{+\infty }\mu \bigl([a,b]\cap \bigl\{ x: f(a)^{1-x}f(b)^{x} \geq r \bigr\} \bigr)\,dr. $$

*Furthermore*, *if* $f(a)< f(b)$, *then*

$$ (C) \int_{[a,b]} f(x)\,d\mu \leq \mu \bigl([a,b]\bigr)f(a)+ \frac{1}{L(f(a),f(b))} \int _{a}^{b}\mu \bigl([x,b]\bigr)f(a)^{1-t}f(b)^{t} \,dx; $$

*and*, *if* $f(a)>f(b)$, *then*

$$ (C) \int_{[a,b]} f(x)\,d\mu \leq \mu \bigl([a,b]\bigr)f(b)- \frac{1}{L(f(a),f(b))} \int _{a}^{b}\mu \bigl([a,x]\bigr)f(a)^{1-t}f(b)^{t} \,dx, $$

*where* $t=\frac{x-a}{b-a}$.

### Proof

Using the log-convexity of *f*, for $x\in [a,b]$, we have

$$ f(x)=f \biggl(\biggl(1-\frac{x-a}{b-a}\biggr)\cdot a+\frac{x-a}{b-a}\cdot b \biggr) \leq f(a)^{1-t}f(b)^{t}=G(x), $$

where $t=\frac{x-a}{b-a}$. We have $G(a)=f(a), G(b)=f(b)$, and

$$ G'(x)= \frac{f(a)^{1-t}f(b)^{t}}{L(f(a),f(b))}. $$

Due to (4) of Lemma 2.1, we have

$$\begin{aligned} (C) \int_{[a,b]} f(x)\,d\mu \leq & (C) \int_{[a,b]} G(x)\,d\mu \\ =& \int_{0}^{+\infty }\mu \bigl([a,b]\cap \bigl\{ x: G(x)\geq r \bigr\} \bigr)\,dr . \end{aligned}$$

If $f(a)< f(b)$, then we have

$$\begin{aligned} & \int_{0}^{+\infty }\mu \bigl([a,b]\cap \bigl\{ x: G(x)\geq r \bigr\} \bigr)\,dr \\ &\quad = \int_{0}^{+\infty }\mu \bigl([a,b]\cap \bigl\{ x: x\geq G^{-1}(r) \bigr\} \bigr)\,dr \\ &\quad = \int_{0}^{f(a)}\mu \bigl([a,b]\bigr)\,dr+ \int_{f(a)}^{f(b)}\mu \bigl(\bigl[G^{-1}(r),b \bigr]\bigr)\,dr \\ &\quad = \mu \bigl([a,b]\bigr)f(a)+\int_{a}^{b}\mu \bigl([x,b]\bigr)G'(x)\,dx . \end{aligned}$$

Conversely, if $f(a)>f(b)$, then we have

$$\begin{aligned} & \int_{0}^{+\infty }\mu \bigl([a,b]\cap \bigl\{ x: G(x)\geq r \bigr\} \bigr)\,dr \\ &\quad = \int_{0}^{+\infty }\mu \bigl([a,b]\cap \bigl\{ x: x\geq G^{-1}(r) \bigr\} \bigr)\,dr \\ &\quad = \int_{0}^{f(b)}\mu \bigl([a,b]\bigr)\,dr+ \int_{f(b)}^{f(a)}\mu \bigl(\bigl[a,G^{-1}(r)\bigr]\bigr)\,dr \\ &\quad = \mu \bigl([a,b]\bigr)f(b)- \int_{a}^{b}\mu \bigl([a,x]\bigr)G'(x)\,dx . \end{aligned}$$

The proof is completed. □

### Example 3.6

Consider the function $f(x)=x2^{x^{2}-2}$ on $\mathbf{R}^{+}$ and the distorted Lebesgue measure $\mu =\mu_{m}$, where $m(x)=x^{2}$ in Examples 3.2 and 3.4. We have $f(1)=1/2$, $f(2)=8$ and, according to Theorem 3.6,

$$\begin{aligned} (C) \int_{[1,2]} x2^{x^{2}-2}\,d\mu_{m} \leq & (C) \int_{[1,2]} 2^{4x-5}\,d\mu_{m} \\ =& \int_{0}^{+\infty }\mu_{m} \bigl([1,2]\cap \bigl\{ x: 2^{4x-5}\geq r \bigr\} \bigr)\,dr \\ =& \int_{0}^{+\infty }\mu_{m} \biggl([1,2]\cap \biggl\{ x: x\geq \frac{1}{4}\bigl( \log_{2}^{r}+5 \bigr) \biggr\} \biggr)\,dr \\ =& \int_{0}^{1/2}\mu_{m}\bigl([1,2]\bigr) \,dr+ \int_{1/2}^{8}\mu_{m}\biggl(\biggl[ \frac{1}{4}\bigl( \log_{2}^{r}+5\bigr),2\biggr] \biggr)\,dr \\ =& \frac{1}{2}+ \int_{1/2}^{8}\biggl(2-\frac{1}{4}\bigl( \log_{2}^{r}+5\bigr)\biggr)^{2}\,dr \\ =& \frac{1}{2}+\frac{1}{16} \int_{1/2}^{8}\bigl(3-\log_{2}^{r} \bigr)^{2}\,dr \\ \approx &1.5906 . \end{aligned}$$

The remainder of this paper will be mainly devoted to Jensen’s inequality of the Choquet integral for log-convex functions.

It is well known that there is a result: if $g: \mathbf{R}^{+}\rightarrow \mathbf{R}^{+}$ is a log-convex function, then *g* is convex. The proof is sketched for the readers’ convenience.

### Proof

We firstly come to show the following inequality (Dieudonne [23]; Kallenberg [24]): Let $x, y\geq 0, 1< p, q<\infty $, and ${\frac{1}{p}+\frac{1}{q}=1}$, then

$$ x^{\frac{1}{p}}y^{\frac{1}{q}}\leq \frac{x}{p}+\frac{y}{q} $$

from the concavity of the logarithm function.

Since *g* is a log-convex function, for all $x, y\geq 0$ and $\lambda \in (0,1)$, we have

$$ g\bigl(\lambda x+(1-\lambda )y\bigr)\leq g(x)^{\lambda } g(y)^{1-\lambda }. $$

Putting $p=\frac{1}{\lambda }, q=\frac{1}{1-\lambda }$, frequently we can get

$$ g(x)^{\lambda } g(y)^{1-\lambda }=g(x)^{\frac{1}{p}} g(y)^{ \frac{1}{q}} \leq \lambda g(x)+(1-\lambda )g(y). $$

So we obtain

$$ g\bigl(\lambda x+(1-\lambda )y\bigr)\leq \lambda g(x)+(1-\lambda )g(y), $$

which means *g* is convex. □

Due to the above result and Lemma 2.3, we can easily show the following theorem.

### Theorem 3.7

(Jensen’s inequality)

*Assume that* $f\in L_{C} ^{+}(\mu )$ *and the non*-*additive measure* *μ* *is concave* (*convex*). *If* $g: \mathbf{R}^{+}\rightarrow \mathbf{R}^{+}$ *is a log*-*convex* (*log*-*concave*) *function*, *then we have*

17 $$ g\biggl((C) \int f \,d\mu \biggr)\leq (\geq ) (C) \int g\circ f \,d\mu . $$

### Proof

Let $y_{0}=(C) \int f \,d\mu $. *g* is log-convex, then *g* is convex. So there exists a line through $y_{0}$, i.e., there exist *a* and *b* such that

$$ a y_{0}+b=g(y_{0})\quad \mbox{and} \quad a y+b\leq g(y) $$

for any $y\in \mathbf{R}$. Therefore, it follows that $a\cdot f(x)+b \leq g\circ f(x)$ for all $x\in X$. When $a>0$, by property $(2)$ in Lemma 2.1, we have $(C) \int a\cdot f \,d\mu =a\cdot (C) \int f \,d\mu $; when $a<0$, we have

$$ (C) \int a\cdot f \,d\mu =-(C) \int (-a)\cdot f \,d\bar{\mu } \geq -(C) \int (-a)\cdot f \,d\mu =a\cdot (C) \int f \,d\mu , $$

because *μ* is concave, which implies $\mu \geq \bar{\mu }$. Then, by Lemma 2.1, we have

$$ g\biggl((C) \int f \,d\mu \biggr)=a\cdot (C) \int f \,d\mu +b \leq (C) \int (a\cdot f+b)\,d\mu \leq (C) \int g\circ f \,d\mu $$

as desired. □

Similarly we shall present the following Jensen inequality for the Choquet integral of the log-convex function in two dimensions.

### Theorem 3.8

(Jensen’s inequality in two dimensions)

*Assume that* $f, h\in L_{C}^{+}(\mu )$ *and the non*-*additive measure* *μ* *is concave* (*convex*). *If* *f*, *h* *are comonotonic and* $g: \mathbf{R}^{+}\rightarrow \mathbf{R}^{+}$ *is a log*-*convex* (*log*-*concave*) *function*, *then we have*

18 $$ g \biggl((C) \int f \,d\mu , (C) \int h \,d\mu \biggr)\leq (\geq ) (C) \int g(f, h)\,d\mu . $$

### Proof

Let $y_{0}=(C) \int f \,d\mu , z_{0}=(C) \int h \,d\mu $. *g* is log-convex, then *g* is convex. So there exists a plane through $(y_{0}, z_{0})$, i.e., there exist *a*, *b*, *c* such that

$$ a y_{0}+b z_{0}+c=g(y_{0}, z_{0}), \quad a y+b z+c\leq g(y, z) $$

for any $y, z\in \mathbf{R}$. Therefore, it follows that $a \cdot f( \omega )+b\cdot h(\omega )+c\leq g(f(\omega ), h(\omega ))$. By Lemma 2.1 and the proof of Theorem 3.7, we have

$$\begin{aligned} g \biggl((C) \int f \,d\mu , (C) \int h \,d\mu \biggr) =&a\cdot \int f \,d\mu +b\cdot (C) \int h \,d\mu +c \\ \leq & \int (a\cdot f+b\cdot h +c)\,d\mu \\ \leq & \int g(f,h)\,d\mu . \end{aligned}$$

Therefore, the conclusion holds. □

### Remark 3.5

1. Here the two discussed Jensen inequalities of the Choquet integral for log-convex functions are valid whenever the considered non-additive measure *μ* is concave, which compares also the articles [9] and [15] where all considered inequalities for Choquet integrals hold whenever *μ* is concave.
  In particular, when the non-additive measure *μ* is a plausibility function or the concave distortion measure $\mu =\mu_{m}$, where *m* is a concave function, both inequalities above hold.
2. In fact, for the Choquet integral of log-convex functions, we might investigate the lower bound, when the considered non-additive measure *μ* is concave, using Jensen’s inequality. We shall provide an easy example below.

### Example 3.7

Consider the function $L(x)=2^{(x+1)^{2}}$ on $\mathbf{R}^{+}$. $L(x)=g\circ f$ is the compound function of $g(u)$ and $f(x)$, where $g(u)=2^{u^{2}}$ and $u=f(x)=x+1$. Evidently $g(u)$ is a log-convex function.

Let the distorted Lebesgue measure $\mu =\mu_{m}$, where $m(x)= \sqrt{x}$. *m* is concave, so $\mu_{m}$ is concave. By Theorem 3.7, we have

$$\begin{aligned} (C) \int_{[0,1]} 2^{(x+1)^{2}}\,d\mu_{m} \geq & 2^{[(C) \int_{[0,1]} (x+1)\,d\mu_{m}]^{2}} \\ =& 2^{[(C)\int_{0}^{1} \frac{x+1}{2\sqrt{1-x}}\,dx]^{2}} = 2^{1.6666^{2}}\approx 6.8571 . \end{aligned}$$

## Conclusions and problems for further investigation

This paper offered the readers both some new results of the upper bound and Jensen’s inequalities of the Choquet integral for the log-convex functions.

- We firstly pointed out that the Hadamard inequality is not valid in the non-additive measure theory, and stated the similar Hadamard inequality of the Choquet integral for monotone log-convex function in the framework of distorted measure.
- We secondly estimated the upper bound of the Choquet integral for the general log-convex functions both in the case of distorted measure and in the case of non-additive measure.
- Finally, we obtained Jensen’s inequality and Jensen’s inequality in two dimensions of the Choquet integral for log-convex functions.

These results are extensions of the Choquet theory.

For further investigation, since we have already had a clear understanding of the upper bound and Jensen’s inequality of the Choquet integral for log-convex functions, it is natural to consider how to use them to estimate unsolvable integrals of this kind. Thus the study of their applications is an interesting topic for further research. On the other hand, we shall continue to explore some other inequalities for the Choquet integral of the log-convex functions and also investigate their applications in some areas.

## Methods

The aim of this paper is to study the upper bound and the lower bound of the Choquet integral for log-convex functions. It is well known that the Hadamard inequality provides the upper (lower) bound for the mean value of a log-convex (log-concave) function, so we want to know whether the Hadamard inequality still holds for the Choquet integral. If the Hadamard inequality is not valid, then how to estimate the upper bound and the lower bound of the Choquet integral for log-convex functions? This is the author’s studying route.

In the paper, we attained the similar Hadamard inequality of the Choquet integral for a monotone log-convex function in the framework of distorted measure by Lemma 2.2 (which permits us to compute the Choquet integral when the non-additive measure is a distorted Lebesgue measure). Then we estimated the upper bound of the Choquet integral for a general log-convex function, respectively, in the case of distorted Lebesgue measure and in the non-additive measure using the basic properties of Choquet integrals. Finally, we studied Jensen’s inequality of the Choquet integral for log-convex functions by Lemma 2.3 (Jensen’s inequality of the Choquet integral).
